# ADAR1p110 promotes hepatocellular carcinoma metastasis via the miR-451a/TUBA1A axis

**DOI:** 10.1016/j.gendis.2025.101770

**Published:** 2025-07-12

**Authors:** Liangzhan Sun, Hui Yang, Pengchao Hu, Jingyi Zheng, Yuyang Du, Shasha Wu, Han Gao, Hao Luo, Yanchen Wang, Fenfen Wang, Jingsong Yan, Xin-Yuan Guan, Yan Li

**Affiliations:** a School of Basic Medical Sciences, Chongqing Medical University, Chongqing 400016, China; b Institute for Data-Driven Tumor Immunology, Chongqing Medical University, Chongqing 400016, China; c Institute of Cancer Research, Shenzhen Bay Laboratory, Shenzhen, Guangdong 518000, China; d Department of Systems Biology, School of Life Sciences, Southern University of Science and Technology, Shenzhen, Guangdong 518000, China; e Department of Oncology, Xiangyang No.1 People's Hospital, Hubei University of Medicine, Xiangyang, Hubei 441000, China; f Shenzhen Hospital, Southern Medical University, Shenzhen, Guangdong 518000, China; g Department of Clinical Oncology, The University of Hong Kong, Hong Kong SAR 999077, China; h State Key Laboratory of Oncology in South China and Collaborative Innovation Center for Cancer Medicine, Sun Yat-sen University Cancer Center, Guangzhou, Guangdong 510275, China

**Keywords:** ADAR1p110, Hepatocellular carcinoma, Metastasis, miR-451a, TUBA1A

## Abstract

ADAR1 is overexpressed in hepatocellular carcinoma (HCC) and has been linked to poor prognosis, metastasis, and recurrence; however, its precise functions and underlying mechanisms, particularly in the context of metastasis, remain inadequately elucidated. This study seeks to elucidate the functions and underlying mechanisms of the most abundantly expressed isoform of ADAR1, ADAR1p110, in the setting of HCC metastasis. In this study, hepatocyte-specific ADAR1p110 knock-in mice and engineered HCC cell lines were utilized to investigate the function of ADAR1p110 *in vivo* and *in vitro*. Genome sequencing, transcriptome sequencing, microRNA sequencing, RNA immunoprecipitation qPCR (RIP-qPCR), and RNA pull-down assays were performed to elucidate the mechanism of ADAR1p110 in HCC. We demonstrated that ADAR1p110 overexpression promotes HCC metastasis by improving the motility of HCC cells. Mechanistically, ADAR1p110 overexpression increases TUBA1A expression, which plays a crucial role in regulating HCC cell motility. At the molecular level, ADAR1p110 suppresses miR-451a biogenesis by competitively binding to pri-miR-451a, thereby preventing its cleavage by the Drosha/DGCR8 complex. Furthermore, we confirmed that TUBA1A is a direct downstream target of miR-451a.

## Introduction

Hepatocellular carcinoma (HCC) remains a leading cause of cancer-related mortality, with metastasis being the primary factor contributing to poor survival outcomes.^1^^,^^2^ Although treatment options for HCC have expanded in recent years, metastatic cases remain largely incurable.^3^ Identifying key regulators and elucidating HCC metastasis mechanisms are essential for developing effective therapeutic strategies.

Cancer metastasis is a multistep process involving local invasion, intravasation, survival in circulation, extravasation, and colonization at distant sites, ultimately leading to secondary tumor formation.^4^ This process is regulated by various molecular mechanisms, including epithelial–mesenchymal transition (EMT) regulators, proteolytic enzymes, adhesion molecules, angiogenesis factors, and immune evasion strategies, which collectively facilitate tumor dissemination and progression.^4^ Our previous study revealed elevated ADAR1 expression in metastatic or recurrent HCC,^5^ yet its specific involvement in metastatic processes and the underlying mechanisms remain unclear. In addition to HCC, ADAR1 has been implicated in metastasis across multiple malignancies, including bladder cancer, colorectal cancer, gastric cancer, and melanoma.^6^, ^7^, ^8^, ^9^ Interestingly, ADAR1 has various effects on metastasis across different cancer types. In HCC, colorectal, and gastric cancers, elevated ADAR1 expression has been associated with enhanced metastasis.^5^, ^6^, ^7^ Conversely, in melanoma and breast cancer, high ADAR1 expression has been linked to metastasis suppression.^8^^,^^10^ Of note, ADAR1 exists in two isoforms: the constitutively expressed ADAR1p110 and the interferon-inducible ADAR1p150. Both isoforms possess a double-stranded RNA binding domain (dsRBD) and a deaminase domain. However, ADAR1p150 uniquely contains a Zalpha domain and a nuclear export signal (NES), enabling it to bind left-handed Z-DNA and Z-RNA.^11^ ADAR1p150 is predominantly localized in the cytoplasm,^12^ while ADAR1p110 is predominantly found in the nucleus but can translocate to the cytoplasm to suppress apoptosis in stressed cells by inhibiting Staufen1-mediated mRNA decay of various anti-apoptotic genes.^11^ The differential predominance and functional differences of the two isoforms, ADAR1p110 and ADAR1p150, in various types of tumors may explain the contradictory roles of ADAR1 in metastases. However, the expression patterns of these isoforms across different cancer types, along with their underlying mechanisms in regulating tumor metastasis, remain poorly understood.

Our recent study revealed that ADAR1p110 is the predominant isoform expressed in HCC and is negatively associated with patient survival, whereas ADAR1p150 does not serve as a reliable prognostic marker for HCC.^13^ Therefore, we hypothesize that the ADAR1-mediated promotion of HCC metastasis is primarily driven by ADAR1p110. To investigate whether ADAR1p110 overexpression affects HCC metastasis, we generated a hepatocyte-specific ADAR1p110 knock-in mouse model. These mice were used to create orthotopic ADAR1p110-overexpressing HCC models through DEN-CCl_4_ induction or crossbreeding with c-Myc mice, enabling us to evaluate the impact of ADAR1p110 overexpression on HCC development and progression. Additionally, by combining *in vivo* experimental lung and liver metastasis models with *in vitro* cell migration and wound-healing assays, we demonstrated that ADAR1p110 overexpression significantly promotes HCC metastasis. Mechanistically, ADAR1p110 facilitates tumor cell motility by up-regulating TUBA1A expression through the inhibition of miR-451a biogenesis. Specifically, ADAR1p110 disrupts miR-451a biogenesis by competitively binding to pri-miR-451a, thereby hindering Drosha-mediated cleavage. TUBA1A is a target gene of miR-451a, which inhibits TUBA1A expression by promoting the degradation of its mRNA.

## Materials and methods

### Ethical approval and clinical samples

Clinical specimens were acquired from patients who underwent hepatectomy at Sun Yat-sen University Cancer Center (Guangzhou, China). The study encompassed a total of 57 pairs of frozen primary tumors and adjacent non-tumor tissues. All patients provided written informed consent, permitting the utilization of their tissues and clinical data for scientific research. Approval for the study was obtained from the Committee for Ethical Review of Research Involving Human Subjects at Sun Yat-sen University Cancer Center (NO. B2024-255-01. Date: 2024.05.10). The Ethics Committee of the Sun Yat-sen University Cancer Center acts on the Helsinki Declaration.

### Animal experiments

All animal experiments conducted in this study were rigorously reviewed and approved by the Institutional Animal Care and Use Committee at the Southern University of Science and Technology (No. SUSTC-JY-2019151. Date: 20190725). The Institutional Animal Care and Use Committee at the Southern University of Science and Technology acts on the Basel Declaration. Adar1p110[flox/flox] knock-in mice (Rosa26^LSL−Adar1p110/LSL−Adar1p110^) were sourced from Cyagen Biosciences (Guangzhou, China), while Albumin-Cre mice and c-Myc mice (H11^LSL−Myc/LSL−Myc^) were procured from the Shanghai Model Organisms Center, Inc. (Shanghai, China). To investigate the function of ADAR1p110 overexpression in HCC, we utilized Alb-Cre transgenic mice crossed with Adar1p110[flox/flox] mice and c-Myc mice to generate a liver-specific Adar1p110 knock-in and c-Myc induced orthotopic liver cancer mouse model.

For DEN/CCl_4_-induced HCC model, on the 12th postnatal day, Wild type (WT) and liver-specific Adar1p110 knock-in mice were intraperitoneally injected with DEN at a 20 mg/kg dose. CCl_4_ was diluted in olive oil at a ratio of 1:5, and at six weeks of age, the mice were injected intraperitoneally once a week with CCl_4_ at a dose of 0.5 μL/g. Mouse body weight was monitored throughout the study, and euthanasia was conducted at 26 weeks. Tumor formation in the livers of both the experimental and control group mice was observed, and corresponding cancerous and non-cancerous tissues were collected for subsequent analysis.

For the lung metastasis assay by tail vein injection, NOD-SCID mice received injections of 1 × 10^6^ luciferase-labeled Huh7 cells at a volume of 0.1 mL. Photos were taken once a week to monitor the metastatic status. Similarly, in the liver metastasis model by intrasplenic injection, NOD-SCID mice were injected with 1 × 10^6^ luciferase-labeled CRL8024 cells at a volume of 0.1 mL. The mice were euthanized, and liver tumor metastases were counted after five weeks.

### Cell lines

HepG2 (HB-8065) and SNU449 (CRL-2234) cells were sourced from the American Type Culture Collection (ATCC) in the United States, while CRL8024 (TCHu119) cells were acquired from the Institute of Virology, Chinese Academy of Sciences, Beijing, China. The 293 T cell line (SCSP-502) was obtained from the Cell Bank affiliated with the Shanghai Institute of Biochemistry and Cell Biology. Authentication via short tandem repeat profiling was conducted on all the cell lines, and regular screening for mycoplasma contamination was performed. The cells were cultured in Dulbecco's modified Eagle's medium or RPMI-1640, supplemented with 10% fetal bovine serum (Gibco) and a 1% penicillin/streptomycin mixture (Gibco), and maintained at 37 °C in a humidified atmosphere containing 5% CO2.

### Transwell migration and wound healing assays

Migration assays were conducted using Chambers (Corning, New York, USA, 8.0 μm pore size) following the manufacturer's instructions (Corning). The count of migrated cells was determined under a 20× objective lens. A total of 1 × 10^6^ cells were seeded into each well of a 6-well plate. When the cell confluency reached the desired level, a 200 μL pipette tip was used to create a scratch wound across the center of each well. The wound areas were measured after 24 and 48 h and analyzed using ImageJ software.

### mRNA, microRNA, and DNA sequencing

For mRNA sequencing, 100 ng of total RNA per sample underwent poly(A) RNA enrichment with magnetic oligo-dT beads. The sequencing library templates were prepared using the cBot cluster generation system (Illumina) with the HiSeq SR Cluster V4 Kit, followed by sequencing on the Illumina HiSeq 2500 using HiSeq SBS V4 Kits. Each sample was sequenced to a depth of approximately 50 million paired-end reads, with an average mapping rate exceeding 95%. The read length was 2 × 150 bp, yielding a total sequencing output of approximately 6 Gbp per sample. Image analysis and base calling were performed via real-time analysis 2.2.38 (RTA) software. The RNA-seq reads were aligned to either the human genome (GRCh38.p13) or the mouse genome (GCF_000001635.26) using HISAT2, with RNA-seq expression quantified as reads per kilobase million (RPKM) using Partek Genomic Suite software (v6.6). Differential gene expression was determined using the Partek GS RNA-seq pipeline, with significantly differentially expressed genes identified based on a false discovery rate (FDR) adjusted *P*-value threshold of less than 0.05 and a fold change cutoff of ±2. Gene Ontology (GO) and Kyoto Encyclopedia of Genes and Genomes (KEGG) term analyses were subsequently performed on the sets of differentially expressed genes.

For microRNA sequencing, libraries were prepared using the NEBNext small RNA library prep kit (New England Biolabs) for cell and tissue samples. Library size selection was performed using the Pippin Prep system (Sage Science) to isolate fragments approximately 147–157 nt in length containing mature miRNAs. Sequencing was performed on an Illumina NextSeq 500 platform, generating approximately 10–15 million reads per sample. Adapter trimming and alignment to the GRCh38 reference genome were performed, with annotation using Ensembl (v84), UCSC (hg38), and miRBase (v21). Normalization and differential miRNA expression analysis were performed using the R packages EdgeR (v3.28.1) and limma (v3.42.2).

Whole Genome Sequencing (WGS) was carried out on the HepG2 cell line using the Illumina HiSeq X platform. Sequencing was performed at an average depth of 30 × coverage. After quality control and alignment to the human reference genome hg38 using the BWA aligner, subsequent steps included duplicate removal, indel realignment, and base quality recalibration using the Genome Analysis Toolkit. SNP and Indel mutations were identified and filtered using Varscan2. Pertinent mutations were selected based on specific criteria, with copy number alteration analysis performed using Control-FREEC.

### Identification of A-to-I editing in RNA

Identifying potential A-to-I editing sites based on A to G (or T to C on the complementary strand) mutations in the RNA and excluding sites harboring variants also present in the corresponding cell's DNA. Annotating identified sites using REDIportal and disregarding any sites absent from the RADAR, DARNE A-to-I editing databases, and inosinome Atlas. The editing ratio of “G” or “C” instead of “A” or “T” is calculated, and those higher than 10% are classified as a hyper-edited site.

### Plasmids, lentivirus production, cell infection and transfection

The wild-type ADAR1p110 isoform, catalytically inactive ADAR1p110 mutant (E617A), and double-strand RNA binding motif lost the function of the ADAR1p110 mutant (3ΔdsRBD) were constructed using the pLenti6 plasmid (Invitrogen, USA). The QuikChange II Site-Directed Mutagenesis Kit (Agilent) was used to construct the above mutants following the manufacturer's instructions. Scrambled control shRNA and sh-ADAR1 were constructed using the pLL3.7 plasmid (Addgene, USA). These plasmids were packaged with the pLenti6/V5 Directional TOPO Expression Kit (Invitrogen) and transfected into 293 T cells. The virus was collected after 48 h and the target cell lines were transfected. Blasticidin (Sigma-Aldrich, Germany) or puromycin (ThermoFisher Scientific, USA) was used to select stable overexpression or knockdown cells. For the CRISPR-Cas9 knockout system, a sgRNA targeting ADAR1p110 was inserted into the PX458 plasmid (Addgene, USA) and transfected into target cells. The following sequences of shRNA, sgRNA, siRNA, and Mimic-miR-451a were used in this study: shADAR1-1 GTGAAGATAACAGTGGG; shADAR1-2 ACTGCGAAGGATAGTATAT; sgADAR1 TCTGTCAAATGCCATATGGG; siTUBA1A GAGCGTCCAACCTATACTA; and Mimic-miR-451a AAACCGUUACCAUUACUGAGUU.

### Quantitative reverse-transcription polymerase chain reaction (qRT‒PCR) and RNA immunoprecipitation

Total RNA was extracted with TRIzol reagent (Applied Biosystems Inc., USA), and complementary DNA (cDNA) was synthesized using the PrimeScript™ RT Reagent Kit (Takara, Dalian, China) following the manufacturer's protocol. qPCR analysis was performed with a TB Green Premix Ex Taq kit (Takara, Dalian, China), and the relative expression of each gene was normalized to that of GAPDH or 18S rRNA and determined using the 2-ΔΔCt method. A miRcute Plus miRNA cDNA First-Strand cDNA Kit (TINGEN, GKR211-02) was used to perform reverse transcription of the first strand cDNA of the miRNA. The RNA immunoprecipitation assay was performed as previously described.^14^ Briefly, ADAR1p110-Flag was overexpressed in ADAR1 knockout cells and were then lysed with RIPA lysis buffer. For each IP, 3 mg of protein lysate and 50 μg anti-FLAG or IgG control antibody were used. RNA was extracted from the IPs and qRT**‒**PCR was performed. The primers used in this study are as follows:

**Table:** Table 1. Primer sequences used in this study

Gene	Forward Primer	Reverse Primer
18s	GGAGTATGGTTGCAAAGCTGA	GGAGTATGGTTGCAAAGCTGA
ADAR1	TCTGTCACATTGGGTTACCT	TGCACTCCCATCTCTTGTC
TUBA1A	TCGATATTGAGCGTCCAACCT	CAAAGGCACGTTTGGCATACA
pre-mir-451a	GAATGGCAAGGAAACCGTTA	TCTGGGTATAGCAAGAGAACCAT
pri-mir-451a	GGAGGACAGGAGAGATGCTG	CCTGAGTTCTCTTCCTGGCA

miR-451a-F: GCGCGAAACCGTTACCATTAC. U6 F: CTCGCTTCGGCAGCACA. Reverse primers for miR-451a and U6 were obtained from the miRcute Plus miRNA qPCR Kit (SYBR Green) (TINGEN, FP411).

### Luciferase reporter assays

A luciferase assay kit (Yeasen, China) was used to perform the luciferase reporter assay following the manufacturer's protocol. Briefly, cells were plated in 6-well plates and then were transfected with 200 nm NC mimics or miR-451a mimics with 1 μg of the WT or MUT 3′UTR plasmid. After 48 h of transfection, the cells were collected and lysed with lysis buffer, and the activities of Firefly and Renilla luciferase in the lysates were measured with a kit. The luminescence was measured with a PerkinElmer EnSpire multimode reader. The results were excluded from the blank value and then normalized to Renilla luciferase activity.

### Flow cytometry

To perform Ki67 staining for flow cytometry analysis, the cell suspensions were prepared and incubated with an Fc blocking antibody (CD16/32, BD Biosciences) for 10 min at 4 °C to prevent non-specific binding. Next, dead cells were labeled using the Zombie NIR Fixable Viability Kit (BioLegend, Cat# 423105) to exclude them from the analysis. After labeling dead cells, they were fixed and permeabilized using the Foxp3/Transcription Factor Staining Buffer Set (Thermo Fisher, Cat# 00-5532-00) for intracellular staining. Following permeabilization, the cells were stained with a Ki67 antibody (1:100, BioLegend, Cat# 151208) to detect proliferating cells. The mixture was incubated for 30 min at 4 °C and then washed thoroughly to remove excess antibody. The Dead Cell Apoptosis Kit with Annexin V Alexa Fluor 488 & Propidium Iodide (PI) (ThermoFisher, Cat# V13241) was used for flow cytometry to measure apoptosis following the manufacturer's protocol.

### RNA pulldown assay and Western blot

An affinity pulldown of biotinylated pri-mir-451a mRNA was conducted to detect protein–RNA complexes as previously described.^15^ The cell lysate was prepared, and a pulldown was conducted to isolate the interacting RNA-binding protein. The interacting RNA-binding proteins were identified using Western blot. The following antibodies were used in this study: GAPDH (1:1000, CST, Cat# 2118s), ADAR1 (1:1000, Abcam, Cat# ab226188), Drosha (1:1000, CST, Cat# 3364 S), and DGCR8 (1:1000, Proteintech, Cat# 10996-1-AP).

### Statistical analysis

Statistical analyses were conducted using GraphPad Prism 9.0. A paired two-tailed Student's *t*-test was employed to compare the mRNA levels of ADAR1 and TUBA1A in paired non-tumor and tumor samples. An unpaired two-tailed Student's *t*-test was employed to compare the number of tumors, migration cells, wound healing area, and relative gene expression between two predefined groups. When the data could be classified into three or more groups, one-way ANOVA with Duncan's multiple comparisons test was employed. *In vivo* metastasis assays were analyzed with the exact binomial test. Kaplan–Meier plots and log-rank tests were utilized to assess differences in overall survival. Pearson's correlation coefficients were employed to examine correlations. Statistical significance was defined as a *P* value less than 0.05.

## Results

### ADAR1p110 promotes HCC metastasis

**Figure 1:**
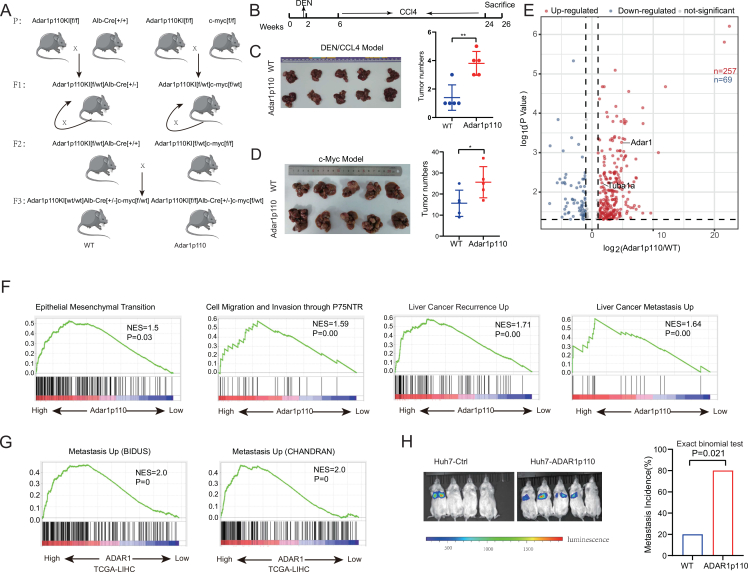
ADAR1p110 promotes HCC metastasis. **(A)** Schematic diagram of breeding liver-specific Adar1p110 knock-in and c-Myc induced orthotopic liver cancer mouse model. **(B)** Schematic diagram of DEN/CCl_4_-induced HCC mouse model. **(C)** Representative image of DEN/CCl_4_-induced mouse HCC and statistical analysis of tumor numbers (*n* = 5). **(D)** Representative image of c-Myc-induced mice HCC and statistical analysis of tumor numbers (*n* = 5). **(E)** Volcanic maps of differentially expressed genes in the c-Myc induced mouse HCC model. Red dots: significantly up-regulated in Adar1p110 knock-in mice. Blue dots: significantly down-regulated in Adar1p110 knock-in mice. **(F)** Results of gene set enrichment analysis (GSEA) of mRNA-seq data from the c-Myc induced mouse HCC model. **(G)** Results of gene set enrichment analysis (GSEA) of TCGA-LIHC mRNA-seq data based on ADAR1 expression. **(H)** Representative images and statistical results of the tail vein injection lung metastasis model (*n* = 5, one mouse in the WT group died due to anesthesia; autopsy confirmed that it had no metastasis). The data are presented as the mean ± SD. *P* values were computed using the unpaired Student's *t*-test (C, D) and Exact binomial test (H). ∗*P* < 0.05, ∗∗*P* < 0.01.

To elucidate the role of ADAR1p110 overexpression in HCC, we developed orthotopic HCC models in WT and hepatocyte-specific Adar1p110 knock-in mice using two distinct approaches: hepatocyte-specific overexpression of c-Myc and DEN/CCl_4_-induced hepatocarcinogenesis (Fig. 1A and B). In both models, the number of tumors was significantly increased in the Adar1p110 overexpression group compared to the WT group (Fig. 1C and D). To investigate the mechanisms underlying the increased tumor burden associated with Adar1p110 overexpression, we performed transcriptome sequencing on c-Myc-driven orthotopic tumor samples and analyzed the results using gene set enrichment analysis (GSEA) (Fig. 1E and F and Table S1). GSEA analysis revealed that multiple pathways associated with tumor metastasis and recurrence, including signaling pathways linked to EMT, cell migration, invasion, liver cancer recurrence, and liver cancer metastasis, were significantly activated in the Adar1p110 overexpression group (Fig. 1F). Consistent with the findings in mouse models, analysis of The Cancer Genome Atlas Liver Hepatocellular Carcinoma (TCGA-LIHC) data revealed enrichment of the liver cancer metastasis pathway in patients with high ADAR1p110 expression (Fig. 1G). The above results suggest that ADAR1p110 may play a significant role in HCC metastasis. However, using our two orthotopic HCC models, we could not determine whether the additional tumors originated from intrahepatic metastasis or *de novo* tumorigenesis. To validate the potential role of ADAR1p110 in promoting HCC metastasis, a lung metastasis model was established by tail vein injection in NOD-SCID mice. The results demonstrated that the overexpression of ADAR1p110 in HCC cells significantly enhances their metastatic potential (Fig. 1H). These *in vivo* experiments confirm that ADAR1p110 contributes to HCC metastasis.

### ADAR1p110 regulates the motility of HCC cancer cells *in vitro*

**Figure 2:**
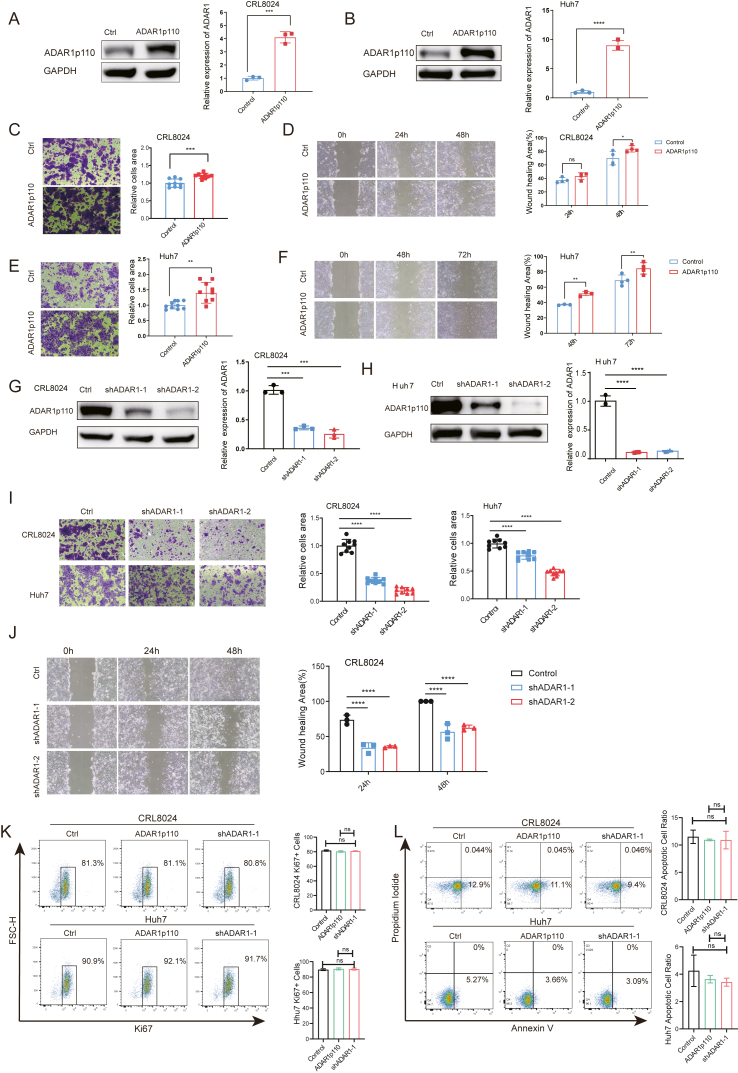
ADAR1p110 regulates the motility of HCC cancer cells *in vitro*. **(A, B)** Ectopic expression of ADAR1p110 in CRL8024 (A) and Huh7 (B) cells was verified by qRT‒PCR and Western blots (*n* = 3). **(C, D)** Representative images and quantification of migration (C) (*n* = 9) and wound healing (D) (*n* = 4) induced by CRL8024 cells. **(E, F)** Representative images and quantification of migration (E) (*n* = 10) and wound healing (F) (*n* = 4) induced by Huh7 cells. **(G, H)** Ectopic knockdown of ADAR1 in CRL8024 (G) and Huh7 (H) cells was verified by qRT‒PCR and Western blots (*n* = 3). **(I)** Representative images and statistical results of the migration in the indicated cells (*n* = 9). **(J)** Representative images and statistical wound healing results in the indicated cells (*n* = 3). **(K)** Proliferation was measured by flow cytometry. Representative images and statistical results of the Ki6 positive PLC8024 and Huh7 cells (*n* = 3). **(L)** Apoptosis was measured by flow cytometry. Representative images and statistical results of PLC8024 and Huh7 apoptosis (*n* = 3). The data are presented as the mean ± SD. *P* values were computed using the unpaired Student's *t*-test (A, B, C, D, E, F) and one-way ANOVA test (G, H, I, J, K, L). ns: not significant. ∗*P* < 0.05, ∗∗*P* < 0.01, ∗∗∗*P* < 0.001, ∗∗∗∗*P* < 0.0001.

Next, we generated HCC cell lines with ADAR1p110 overexpression using lentiviral transduction (Fig. 2A and B). Transwell migration assays and wound-healing experiments demonstrated that ADAR1p110 overexpression significantly enhances the migratory and wound-healing abilities of HCC cells (Fig. 2C–F). Conversely, ADAR1p110 knockdown markedly impaired these capacities (Fig. 2G–J). These findings confirm that ADAR1p110 directly modulates the motility of liver cancer cells.

To further examine the role of ADAR1p110, we assessed its impact on cancer cell proliferation and apoptosis. Under normal conditions, neither overexpression nor knockdown of ADAR1p110 significantly affected Ki67 expression, indicating that ADAR1p110 does not influence cancer cell proliferation (Fig. 2K). Similarly, apoptosis assays revealed that ADAR1p110 expression levels had no significant effect on apoptosis (Fig. 2L). Collectively, these findings indicate that ADAR1p110 primarily promotes cancer progression by facilitating tumor cell metastasis rather than regulating proliferation or apoptosis.

### ADAR1p110 expression is positively correlated with TUBA1A

**Figure 3:**
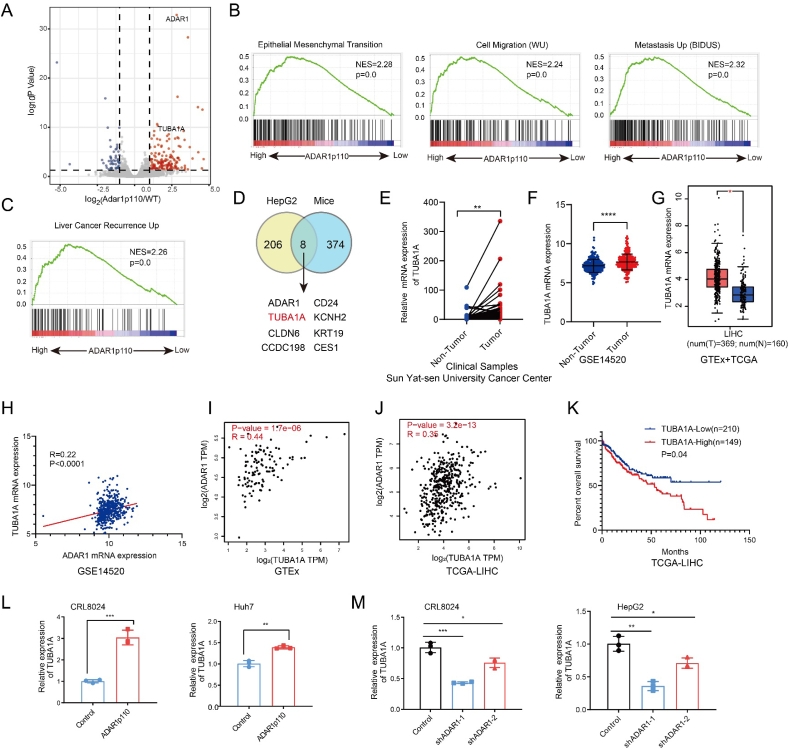
The expression of ADAR1p110 correlated with that of TUBA1A. **(A)** Volcanic maps of differentially expressed genes in genetically engineered human HCC cells. Red dots: significantly up-regulated in ADAR1p110-overexpressing cells. Blue dots: significantly down-regulated in ADAR1p110-overexpressing cells. **(B, C)** Results of gene set enrichment analysis (GSEA) of mRNA seq data from genetically engineered human HCC cells. **(D)** Venn diagram showing consistently altered genes in human and mouse models with ADAR1p110 overexpression. **(E–G**) The expression level of TUBA1A in tumor and non-tumor tissues from Sun Yat-sen University Cancer Center (*n* = 57) (E), GSE14520 (F, Tumor *n* = 247, Non-tumor *n* = 239), and TCGA-LIHC combined with GTEx (G, Tumor *n* = 369, Non-tumor *n* = 160). **(H**–**J)** ADAR1 mRNA expression positively correlates with TUBA1A expression across multiple datasets. GSE14520 (H, *n* = 488); GTEx (I, *n* = 97); TCGA-LIHC (J, *n* = 423). **(K)** Kaplan–Meier overall survival curves of TCGA-LIHC patients with low or high TUBA1A expression (TUBA1A-High *n* = 149, TUBA1A-Low *n* = 210). **(L, M)** The expression level of TUBA1A in ADAR1p110 overexpression (L) or ADAR1p110 knockdown (M) cells was verified by qRT‒PCR (*n* = 3). The data are presented as the mean ± SD. *P* values were computed using the unpaired Student's *t*-test (D, E, F, G, L), one-way ANOVA test (M), Pearson's correlation test (H, I, J), and log-rank tests (K). ns: not significant. ∗*P* < 0.05, ∗∗*P* < 0.01, ∗∗∗*P* < 0.001, ∗∗∗∗*P* < 0.0001.

To elucidate the mechanisms by which ADAR1p110 regulates tumor cell migration, we conducted mRNA sequencing on HCC cell lines overexpressing ADAR1p110 and the control (Fig. 3A and Table S2). GSEA revealed that, consistent with findings from murine HCC tissues, signaling pathways associated with epithelial–mesenchymal transition (EMT), cell migration, metastasis, and liver cancer recurrence were enriched in cells with high ADAR1p110 expression (Fig. 3B and C). By comparing the sequencing data of murine HCC and human cell lines overexpressing ADAR1p110, we found that ADAR, CD24, TUBA1A, KCNH2, CLDN6, KRT18, CCDC198, and CES1 were significantly changed in both the human and murine ADAR1p110-overexpressing groups (Figure 1, Figure 3D). Notably, among these significantly altered genes, previous studies have indicated that TUBA1A plays a critical role in tumor metastasis.^16^ Furthermore, through the analysis of clinical samples from Sun Yat-sen University Cancer Center, GSE14520, GTEx-Liver, and TCGA-LIHC data, we observed that TUBA1A was highly expressed in liver cancer tissues (Fig. 3E–G) and was positively correlated with the expression of ADAR (Fig. 3H–J). Additionally, our analysis of TCGA-LIHC data revealed that patients with high expression of TUBA1A in liver cancer have a poorer prognosis (Fig. 3K). Furthermore, in our own constructed cell lines, we found that the overexpression of ADAR1p110 can increase the expression of TUBA1A, while the knockdown of ADAR1p110 can suppress the expression of TUBA1A (Fig. 3L and M). Based on these findings, we propose that ADAR1p110 modulates cancer cell motility by regulating TUBA1A expression, thereby facilitating HCC metastasis.

### TUBA1A plays a key role in regulating HCC cancer cell motility

**Figure 4:**
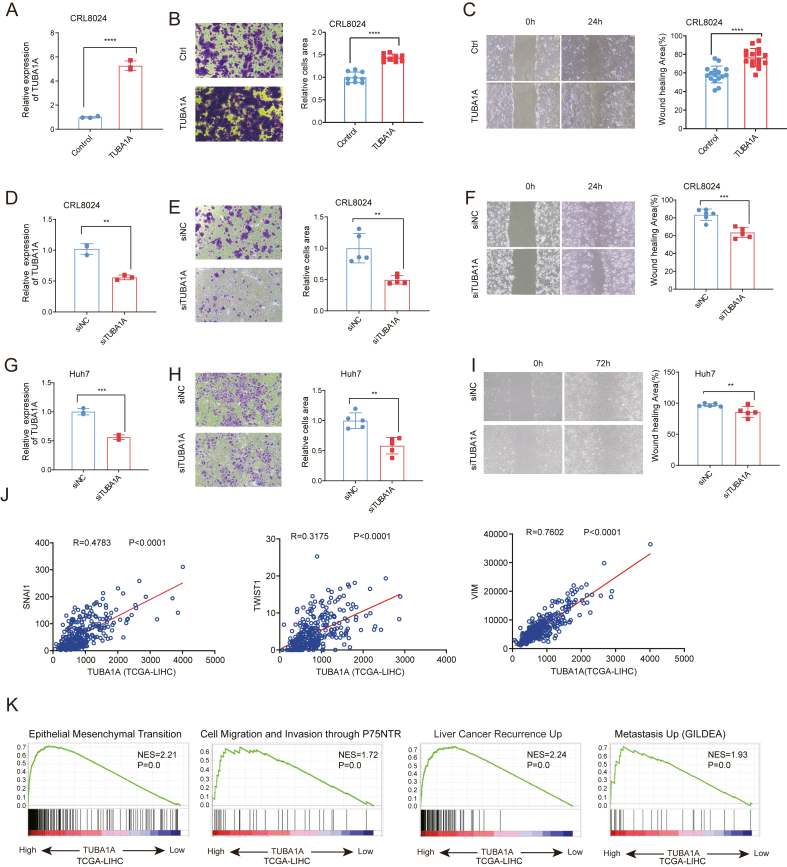
TUBA1A plays a key role in regulating HCC cell motility. **(A)** Ectopic expression of TUBA1A in CRL8024 was verified by qRT‒PCR (*n* = 3). **(B, C)** Representative images and quantification of migration (B) (*n* = 9) and wound healing (C) (*n* = 16) induced by the indicated CRL8024 cells. **(D)** The silencing efficacy of siTUBA1A in CRL8024 was quantified by qRT‒PCR (*n* = 3). **(E, F)** Representative images and quantification of migration (E) (*n* = 5) and wound healing (F) (*n* = 6) induced by the indicated CRL8024 cells. **(G)** The silencing efficacy of siTUBA1A in Huh7 was quantified by qRT‒PCR (*n* = 3). **(H, I)** Representative images and quantification of migration (H) (*n* = 5) and wound healing (I) (*n* = 5) induced by the indicated CRL8024 cells. **(J)** The mRNA expression of TUBA1A was positively correlated with that of SNAI1, TWIST1, and VIM in the TCGA-LIHC dataset (*n* = 423). **(K)** Results of gene set enrichment analysis (GSEA) of TCGA-LIHC mRNA-seq data based on TUBA1A expression. The data are presented as the mean ± SD. *P* values were computed using the unpaired Student's *t*-test (A–I), and Pearson's correlation test (J). ∗*P* < 0.05, ∗∗*P* < 0.01, ∗∗∗*P* < 0.001, ∗∗∗∗*P* < 0.0001.

The role of TUBA1A in promoting the motility of HCC cells has not been addressed previously. In this study, we established a liver cancer cell line with TUBA1A overexpression and observed that elevated TUBA1A levels significantly enhanced the migratory capacity of liver cancer cells, as demonstrated by transwell and wound healing assays (Fig. 4A–C). Conversely, TUBA1A knockdown markedly impaired cell motility (Fig. 4D–I). Additionally, TCGA-LIHC data analysis revealed a positive correlation between TUBA1A expression and key epithelial–mesenchymal transition (EMT) markers, including VIM, TWIST, and SNAI1 (Fig. 4J). GSEA analysis based on TCGA-LIHC data further demonstrated significant enrichment of pathways associated with EMT, cell migration and invasion, liver cancer recurrence, and metastasis in patients with high TUBA1A expression (Fig. 4K). Collectively, these findings highlight the critical role of TUBA1A in regulating liver cancer cell motility and its potential contribution to HCC metastasis.

### ADAR1p110 regulates the expression of TUBA1A by inhibiting the expression of miR-451a

**Figure 5:**
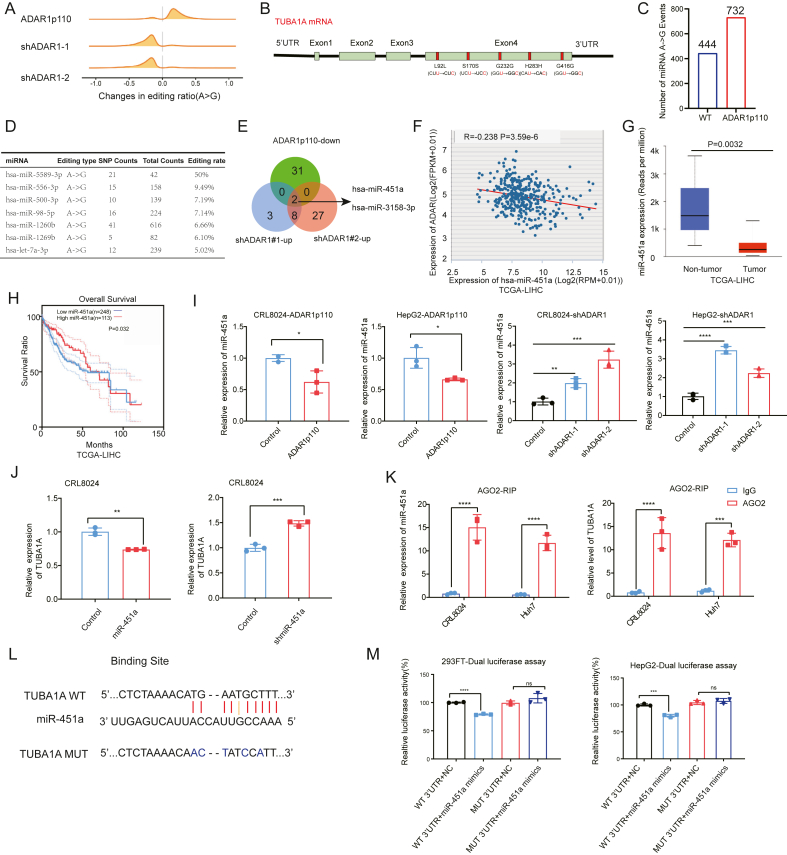
ADAR1p110 regulates the expression of TUBA1A by inhibiting the expression of miR-451a. **(A)** Changes of RNA editing ratio after ADAR1p110 overexpression or knockdown. **(B)** Schematic diagram of TUBA1A editing. **(C)** Statistics of microRNA editing events in WT and ADAR1p110-overexpressing HCC cells. **(D)** miRNAs with editing counts greater than 5 and editing ratio greater than 5% in ADAR1p110-overexpressing or ADAR1p110 knockdown cells. **(E)** miRNAs were down-regulated after ADAR1p110 overexpression and up-regulated after ADAR1 knockdown. **(F)** The mRNA expression of TUBA1A was negatively correlated with that of miR-451a analysis based on the TCGA-LIHC dataset (*n* = 370). **(G)** The expression level of miR-451a in tumor and non-tumor tissues from the TCGA-LIHC dataset (Tumor *n* = 369, Non-tumor *n* = 49). **(H)** Kaplan–Meier overall survival curves of TCGA-LIHC patients with low or high expressed miR-451a (Low miR-451a *n* = 248, High miR-451a *n* = 113). **(I)** The expression of miR-451a in the indicated cells was verified by qRT‒PCR (*n* = 3). **(J)** The expression of TUBA1A in the indicated cells was verified by qRT‒PCR (*n* = 3). **(K)** The relative expression of miR-451a and TUBA1A in anti-AGO2 antibody precipitated RNA (*n* = 3). **(L)** Schematic diagram of miR-451a binding with the WT and mutated 3′-UTR of TUBA1A. **(M)** Luciferase activities of TUBA1A-WT or TUBA1A-MUT were determined in the presence of the NC mimic or the miR-451a mimic (*n* = 3). The data are presented as the mean ± SD. *P* values were computed using the unpaired Student's *t*-test (G, I, J, K, M), one-way ANOVA test (I), Pearson's correlation test (F), and log-rank tests (H). ns: not significant. ∗*P* < 0.05, ∗∗*P* < 0.01, ∗∗∗*P* < 0.001, ∗∗∗∗*P* < 0.0001.

Next, we endeavored to elucidate the mechanisms underlying the ADAR1p110-mediated regulation of TUBA1A expression. Given that ADAR1 is a key RNA-editing enzyme that catalyzes the conversion of adenosine (A) to inosine (I) in double-stranded RNA (dsRNA), we first explored the impact of RNA editing on TUBA1A by comparing mRNA sequencing data with genome sequencing data. Following ADAR1p110 overexpression, the global mRNA editing ratio increased, whereas ADAR1 knockdown resulted in a decreased global mRNA editing ratio (Fig. 5A and Table S3). Analysis of TUBA1A mRNA revealed no discernible modifications in editing sites within the 3′-UTR, 5′-UTR, or intronic regions, but identified four altered editing sites within exon 4 (Fig. 5B). Yet, these changes in editing sites were synonymous mutations and did not alter the amino acid sequence. Given the modulation of microRNA (miRNA) biogenesis, maturation or sequence alterations have emerged as a pivotal mechanism through which ADAR1 exerts its regulatory influence on gene expression. We performed miRNA sequencing, analyzed miRNA editing events, and found that ADAR1p110 overexpression markedly increased the number of edited miRNAs, escalating from 444 to 732 (Fig. 5C and Table S4). We selected miRNAs with editing counts greater than 5 and editing ratios greater than 5% and analyzed them using TarBase and TargetScan, but found that these miRNAs could not regulate the expression of TUBA1A (Fig. 5D and Table S4). The above experimental data indicate that ADAR1p110 does not regulate TUBA1A expression by altering the TUBA1A mRNA or miRNA sequence through its editing function.

Modulating gene expression through the regulation of miRNA expression levels represents another important mechanism by which ADAR1 controls gene expression.^17^ Thus, we analyzed the microRNA expression levels using microRNA-seq data from cells with ADAR1p110 overexpression, knockdown, and control. We identified two microRNAs regulated by ADAR1p110 in HCC: miR-451a and miR-3158-3p, which were down-regulated after ADAR1p110 overexpression and up-regulated after ADAR1 knockdown (Fig. 5E). Analysis of TCGA data revealed a negative correlation between miR-451a expression and ADAR1 (Fig. 5F). The expression of miR-451a was significantly lower in liver cancer tissues than in adjacent normal tissues (Fig. 5G), and patients with high miR-451a expression had prolonged overall survival (Fig. 5H). However, as miR-3158-3p showed no difference in expression between tumor and adjacent non-tumor tissues and had no impact on patient survival, we focused our subsequent studies on miR-451a. Furthermore, we confirmed that the overexpression of ADAR1p110 can reduce miR-451a expression, while knockdown of ADAR1p110 can increase miR-451a expression in our constructed cell lines (Fig. 5I). The above experimental results indicate that ADAR1p110 can regulate the expression of miR-451a.

Furthermore, the overexpression of miR-451a reduced TUBA1A expression, and the knockdown of miR-451a increased TUBA1A expression (Fig. 5J). To verify whether miR-451a can target and degrade TUBA1A, we performed an RNA immunoprecipitation (RIP) assay using an anti-AGO2 antibody. We found that both miR-451a and TUBA1A can be precipitated by the anti-AGO2 antibody simultaneously (Fig. 5K), proving the direct interaction between miR-451a and TUBA1A. Additionally, we predict the binding site of miR-451a on the TUBA1A 3′-UTR with miRanda^18^ and constructed wild-type and mutant luciferase reporter plasmids for the TUBA1A 3′-UTR (Fig. 5L). Our results showed that the miR-451a mimic significantly decreased the intensity of wild-type TUBA1A 3′-UTR luciferase, with no effect on the intensity of mutant TUBA1A 3′-UTR luciferase (Fig. 5M). These results demonstrate that TUBA1A is a target gene of miR-451a and that miR-451a can down-regulate TUBA1A expression by binding to its 3′-UTR.

### ADAR1p110 inhibits the biogenesis of miR-451a by precluding the binding of the Drosha/DGCR8 complex to Pri-mir-451a

**Figure 6:**
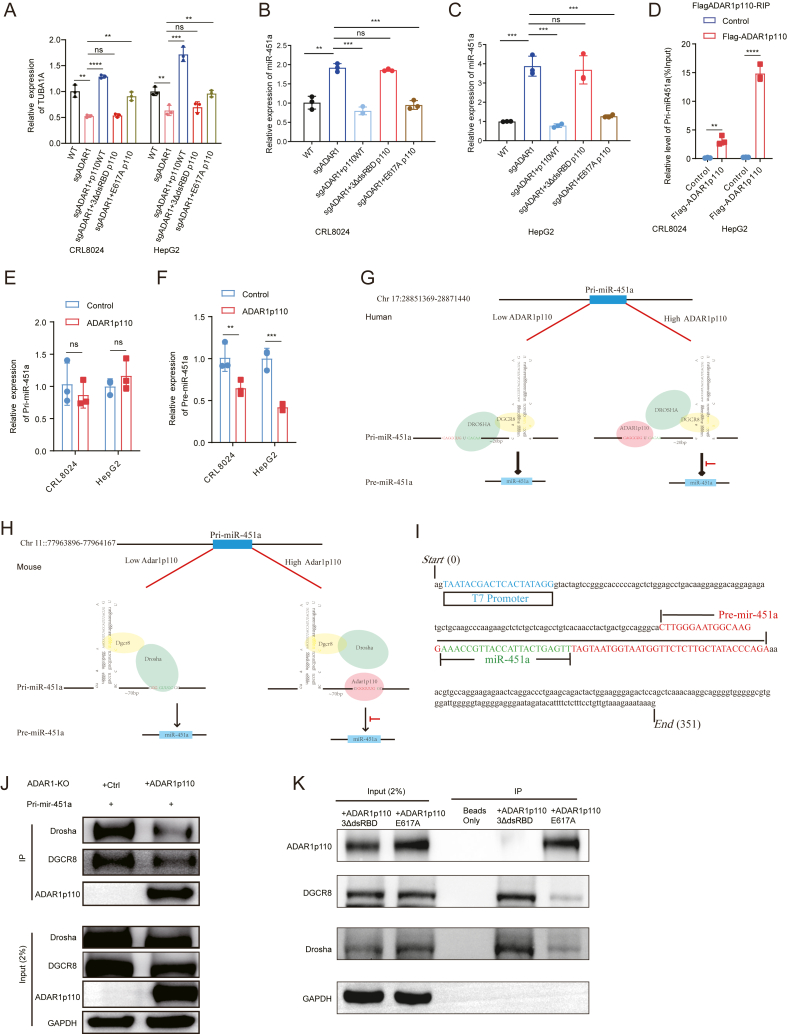
ADAR1p110 inhibits the biogenesis of miR-451a by precluding the binding of Drosha to Pri-miR-451a. **(A)** The relative expression of TUBA1A in the indicated cells was verified by qRT‒PCR (*n* = 3). **(B, C)** The relative expression of miR-451a in the indicated cells was verified by qRT‒PCR (*n* = 3). **(D)** The relative expression of Pri-miR-451a in anti-FLAG antibody precipitated RNA (*n* = 3). **(E, F)** The relative expression of Pri-miR-451a (E) and Pre-miR-451a (F) in the indicated cells (*n* = 3). **(G, H)** Schematic diagram of ADAR1p110 and Drosha binding with Pri-miR-451a. **(I)** Schematic diagram of the *in vitro* transcription of Pre-miR-451a. **(J, K)** Proteins pulled down with Pri-miR-451a were confirmed by Western blot. The data are presented as the mean ± SD. *P* values were computed using the one-way ANOVA test (A–C) and unpaired Student's t-test (D–F). ns: not significant. ∗∗*P* < 0.01, ∗∗∗*P* < 0.001, ∗∗∗∗*P* < 0.0001.

ADAR1 can affect the expression level of microRNAs through RNA editing, RNA-binding, or as a scaffold independent of RNA editing or binding function.^17^ To investigate how ADAR1p110 regulates the level of miR-451a expression, we constructed ADAR1 knockout cell lines using the CRISPR-Cas9 system, separately replenished them with wild-type ADAR1p110, an enzymatically inactive mutation (E617A), and double-strand RNA-binding motif loss-of-function mutation (3ΔdsRBD) ADAR1p110 mutants, and measured the expression level of miR-451a. We found that both wild-type and enzymatically inactive mutant ADAR1p110 could rescue the decrease in TUBA1A and the increase in miR-451a caused by ADAR1 knockout, while the double-strand RNA-binding motif lost function mutation (3ΔdsRBD) variant could not rescue (Fig. 6A–C). These results indicate that ADAR1p110 regulates TUBA1A and miR-451a in a manner dependent on its double-strand RNA-binding rather than RNA-editing function. Subsequently, we overexpressed ADAR1p110-FLAG in ADAR1 knockout cells and performed and RIP Assay using an anti-FLAG antibody to detect ADAR1p110-binding RNA via qPCR, revealing that ADAR1p110 can bind to pri-mir-451a (Fig. 6D). Simultaneously, we measured the expression of pri-mir-451a and pre-mir-451a and found that overexpression of ADAR1p110 did not affect pri-mir-451a expression, but significantly decreased pre-mir-451a expression (Fig. 6E and F). These results confirm that ADAR1p110 can influence the biogenesis of miR-451a by affecting the biogenesis from pri-mir-451a to pre-mir-451a. We analyzed human and mouse pri-mir-451a sequences and found that ADAR1p110 and the Drosha/DGCR8 complex compete for binding to pri-mir-451a. In human pri-mir-451a, the binding sites of ADAR1p110 are very close to Drosha/DGCR8 binding sites, with only one base separating them. Meanwhile, in mice, they overlap by 4 bp in the pri-mir-451a. Thus, we hypothesize that ADAR1p110 affects the expression of miR-451a by precluding the binding of Drosha/DGCR8 to pri-mir-451a, influencing its cleavage and therefore affecting the biogenesis of miR-451a (Fig. 6G and H). To verify our hypothesis, we synthesized biotin-labeled pri-mir-451a using an *in vitro* transcription method and performed an RNA pull-down assay (Fig. 6I). The proteins bound to pri-mir-451a were then validated by Western blot analysis. Our experimental results indicate that when ADAR1p110 binds to pri-mir-451a, it can reduce the binding of Drosha/DGCR8 (Fig. 6J). Furthermore, to determine whether the double-stranded RNA-binding domain is required for this competitive interaction, we repeated the RNA pull-down assay using the 3ΔdsRBD mutant and the catalytically inactive ADAR1p110 mutant (E617A). As shown in Figure 6K, while the catalytically inactive ADAR1p110 mutant (E617A) significantly reduced the binding of Drosha and DGCR8 to pri-miR-451a, the 3ΔdsRBD variant failed to displace Drosha and DGCR8 from pri-miR-451a, indicating that loss of RNA-binding capacity abolishes competitive inhibition. This result further confirms that the RNA-binding function of ADAR1p110 is essential for its ability to suppress of miR-451a biogenesis through steric hindrance.

The above results indicate that ADAR1p110 can utilize its mRNA-binding function, rather than its editing function, to create steric hindrance. By reducing the binding of Drosha/DGCR8 to pri-mir-451a, it decreases the biogenesis of pre-mir-451a, ultimately leading to a down-regulation of miR-451a.

### miR-451a abolishes ADAR1p110-mediated HCC metastasis

**Figure 7:**
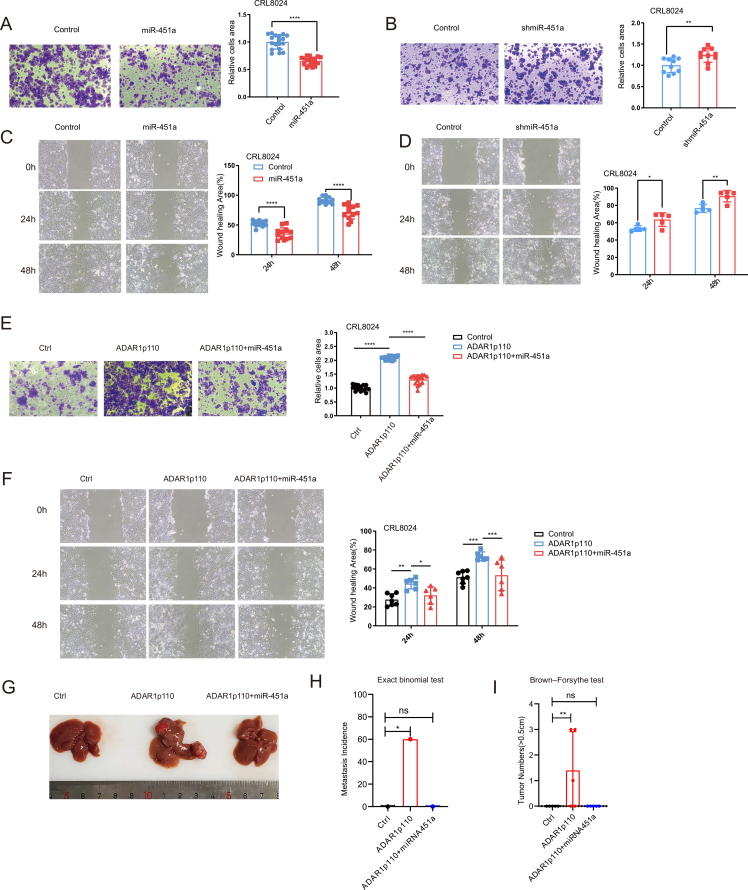
miR-451a abolishes ADAR1p110-mediated HCC metastasis. **(A, B)** Representative images and quantification of migration induced by miR-451a overexpression (A) (*n* = 18) or knockdown (B) (*n* = 10) cells. **(C, D)** Representative images and quantification of wound healing induced by miR-451a overexpression (C) (*n* = 12) or knockdown (D) (*n* = 5) cells. **(E)** Representative images and quantification of migration induced by the indicated CRL8024 cells (*n* = 15). **(F)** Representative images and quantification of wound healing induced by indicated CRL8024 cells (*n* = 6). **(G**–**I)** Representative image (G) and quantification of the metastasis rates (H) and tumor numbers (I) of the spleen injection liver metastasis mouse model. The data are presented as mean ± SD. *P* values were computed using the unpaired Student's *t*-test (A–D), one-way ANOVA test (E, F), Exact binomial test (H), and Brown–Forsythe test (I). ns: not significant. ∗*P* < 0.05, ∗∗*P* < 0.01, ∗∗∗*P* < 0.001, ∗∗∗∗*P* < 0.0001.

Subsequently, we investigated the function of miR-451a and its potential involvement in the ADAR1p110-TUBA1A axis in regulating HCC metastasis. In HCC cell lines, the overexpression of miR-451a significantly inhibited tumor cell migration (Fig. 7A), while the down-regulation of miR-451a markedly enhanced tumor cell migration capability (Fig. 7B). Consistently, in wound healing experiments, we observed that miR-451a overexpression significantly delays wound healing, whereas miR-451a down-regulation accelerates tumor cell wound healing (Fig. 7C and D). To confirm that ADAR1p110 regulates HCC metastasis through the miR-451a/TUBA1A axis, we overexpressed miR-451a in ADAR1p110-overexpressing HCC cells and found that miR-451a overexpression suppresses the pro-metastatic effect induced by ADAR1p110 overexpression (Fig. 7E). Wound healing experiments yielded consistent results (Fig. 7F). Furthermore, we validated these findings using *in vivo* animal experiments. We established a liver metastasis model by intrasplenic injection and found that, consistent with findings in the tail vein model, the overexpression of ADAR1p110 significantly promotes tumor cell metastasis. However, when we introduced miR-451a into cells overexpressing ADAR1p110, it significantly inhibited the metastasis induced by ADAR1p110 overexpression (Fig. 7G–I). These results indicate that ADAR1p110 controls HCC metastasis through the miR-451a/TUBA1A axis.

## Discussion

Although most cancer-related deaths are attributed to metastasis, our understanding of this process and the development of effective treatment strategies remain insufficient.^4^ Herein, we found a novel and conserved mechanism by which the ADAR1p110 isoform binds directly to pri-miR-451a via its double-stranded RNA-binding domain, thereby competing with the Drosha/DGCR8 complex and inhibiting pri-miR-451a processing into pre-miR-451a. This suppression leads to reduced levels of mature miR-451a, which in turn relieves its post-transcriptional repression on TUBA1A. The up-regulation of TUBA1A enhances HCC cell motility and promotes metastasis both *in vitro* and *in vivo*.

In this study, TUBA1A was identified as a conserved downstream target of ADAR1p110. Notably, the editing substrates and functional mechanisms of ADAR1 exhibit substantial variability across different cell types and organisms, making the identification of conserved targets across species particularly challenging. This suggests that such conserved targets may play fundamental and biologically significant roles. Microtubules formed by the polymerization of α-/β-tubulin (TUBA/TUBB) dimers are key components of the cytoskeleton of eukaryotic cells and control a variety of cellular processes, such as cell shape, cell motility, and intracellular trafficking in most cell types.^19^ Nine α-tubulin (TUBA) isoforms have been identified in humans, and TUBA1A has been reported to be up-regulated and associated with metastasis in gastric cancer and neuroblastoma.^20^^,^^21^ However, the role of TUBA1A in HCC is still elusive. In this study, we found that TUBA1A promotes HCC metastasis by improving the motility of cancer cells and that knocking down TUBA1A significantly inhibits HCC cancer motility. Notably, TUBA1A plays a crucial role in the assembly and stability of microtubules, which are essential for cell division.^22^ Microtubules form the mitotic spindle, a structure required for accurate chromosome segregation during mitosis, thereby impacting cell proliferation.^22^ However, we observed that ADAR1p110 up-regulated TUBA1A without affecting cell proliferation. This discrepancy may be related to the extent of changes in TUBA1A expression or may depend on the cellular context.

ADAR1 has been shown to influence the genesis, maturation, function, and target interactions of microRNAs in both editing-dependent and editing-independent manner.^17^ In this study, we found that ADAR1p110 inhibits the biogenesis of miR-451a by disturbing the transition from pri-mir-451a to pre-mir-451a. The pri-miRNA transcript is cleaved by the nuclear Drosha/DGCR8 complex to generate the precursor (pre-miRNA), which is then exported to the cytoplasm and further processed by Dicer.^23^ There is considerable controversy regarding the interaction between ADAR1 and the Drosha/DGCR8 complex. A previous study pointed out that ADAR1 interacts with DROSHA and DGCR8 in the nucleus and potentially outperforms DGCR8 in binding to primary miRNAs, thereby increasing the expression of mature miRNA.^12^ However, Nemlich et al pointed out that ADAR1 and Drosha can competitively bind to DGCR8 and that the formation of the ADAR1-DGCR8 complex leads to a reduction in the DGCR8-Drosha interaction, thereby slowing the processing from pri-miRNA to pre-miRNA.^24^ Moreover, another study indicated that ADAR1 can directly bind to Drosha and promote the degradation of the Drosha protein, thereby inhibiting the processing from pri-miRNA to pre-miRNA.^25^ Of note, the above studies validated the interaction of ADAR1 with DGCR8 or Drosha via co-immunoprecipitation without discriminating between the isoforms ADAR1p110 and ADAR1p150. Moreover, prior studies have predominantly explored the interactions among ADAR1, DGCR8, and Drosha in RNA-dependent and single-stranded RNA (ssRNA)-independent contexts without investigating how these three proteins interact with specific pri-miRNA sequences. Herein, we performed RNA pull-down experiments using biotin-labeled pri-mir-451a in ADAR knockout cell lines complemented with ADAR1p110. This approach allows us to investigate more precisely the mechanisms by which ADAR1p110, DGCR8, and Drosha influence pri-mir-451a. We found that the binding sites of ADAR1p110 competed with the binding sites of Drosha/GDCR8 in pri-mir-451a. Hence, ADAR1p110 can prevent Drosha cleavage of pri-mir-451a, resulting in the suppression of miR-451a biogenesis.

In HCC, miR-451a has been reported to suppress metastasis through multiple mechanisms. Specifically, it inhibits EMT by targeting YWHAZ, restricts cancer cell migration by down-regulating ATF2, and suppresses both migration/invasion and *in vivo* metastasis by targeting c-Myc.^26^, ^27^, ^28^ Additionally, exosomal miR-451a inhibits HCC metastasis by inducing endothelial cell apoptosis by targeting LPIN1.^29^ In this study, we found that TUBA1A is a novel and conserved target of miR-451a that regulates the motility of HCC cells. Cancer cell motility plays a crucial role in the processes of invading surrounding tissues, entering the circulatory system, and extravasation.^4^ Inhibiting motility regulators can prevent tumor dissemination, offering potential therapeutic strategies for limiting metastatic progression.^30^ The identification of the ADAR1p110/miR-451a/TUBA1A axis offers novel insights for the development of targeted therapies aimed at inhibiting metastasis.

## CRediT authorship contribution statement

**Liangzhan Sun:** Investigation, Writing – original draft, Data curation, Writing – review & editing, Formal analysis, Software, Conceptualization. **Hui Yang:** Writing – original draft, Software, Conceptualization, Writing – review & editing, Data curation, Formal analysis, Investigation. **Pengchao Hu:** Data curation, Investigation. **Jingyi Zheng:** Investigation, Data curation. **Yuyang Du:** Data curation, Investigation. **Shasha Wu:** Investigation, Data curation. **Han Gao:** Data curation, Investigation. **Hao Luo:** Investigation, Data curation. **Yanchen Wang:** Investigation, Data curation. **Fenfen Wang:** Data curation, Investigation. **Jingsong Yan:** Investigation, Data curation. **Xin-Yuan Guan:** Conceptualization, Resources. **Yan Li:** Funding acquisition, Supervision, Resources, Writing – review & editing, Conceptualization.

## Data availability

All the data generated or analyzed during this study are available within this published article and its Supplementary Online Materials.

Public Datasets: RNA expression data of ADAR1 and TUBA1A were obtained from the TCGA database (https://portal.gdc.cancer.gov/), GTEx database (https://www.gtexportal.org/home/), and GEO dataset GSE14520 (https://www.ncbi.nlm.nih.gov/geo/query/acc.cgi acc = GSE14520).

Statistical graphs shown in Figure 3G–I, and J were generated using the GEPIA2 web tool (http://gepia2.cancer-pku.cn), those in Figure 5F and G using the ENCORI platform (https://rnasysu.com/encori/index.php), and those in Figure 5H using the UALCAN portal (https://ualcan.path.uab.edu/).

## Funding

This work was supported by the 10.13039/501100001809National Natural Science Foundation of China awarded to Y. Li (No. 82372768, 82073127); 10.13039/501100003453Guangdong Provincial Natural Science Fund awarded to Y. Li (China) (No. 2022A1515012284); and Shenzhen Science and Technology Innovation grant awarded to Y. Li (China) (No. JCYJ20220530115204011).

## Conflict of interests

The authors declare no competing interests.

## Appendix A Supplementary data

Table S1 Differentially expressed genes from transcriptome sequencing of mouse HCC tumors (Fig. 1E). Table S1

Table S2 mRNA-seq data from human HCC HepG2 cells (Fig. 3A). Table S2

Table S3 Global and miRNA-specific RNA editing analysis (Fig. 5A). Table S3

Table S4 miRNA expression profiles used in differential expression analyses. Table S4

Table S5 Raw numerical data for all bar graphs, box plots, scatter plots, and survival curves across Figure 1, Figure 2, Figure 3, Figure 4, Figure 5, Figure 6, Figure 7, including grouping information, raw values, statistical tests, and exact p-values. Table S5

Table S6 RNA-seq expression values of representative genes (e.g., ADAR1, TUBA1A, VIM, TWIST, SNAI1) extracted from TCGA-LIHC and GSE14520, along with sample IDs and tissue annotations to facilitate independent validation. All the data are provided in editable formats to promote reproducibility and ensure transparency. Readers can re-download public datasets from the original sources as listed. Table S6

Supplementary Figures Uncropped Western blot images for all panels presented in the main text are provided in Fig. S1 (Fig. 2A, B, G, and H) and S2 (Fig. 6J and K). Supplementary Figures
