# Supplementary figures and images for: ADAR1p110 promotes hepatocellular carcinoma metastasis via the miR-451a/TUBA1A axis
Source: Genes Dis. 2025 Jul 12;13(5):101770. doi: 10.1016/j.gendis.2025.101770 (PMC13254589; doi:10.1016/j.gendis.2025.101770)

Figure 2A

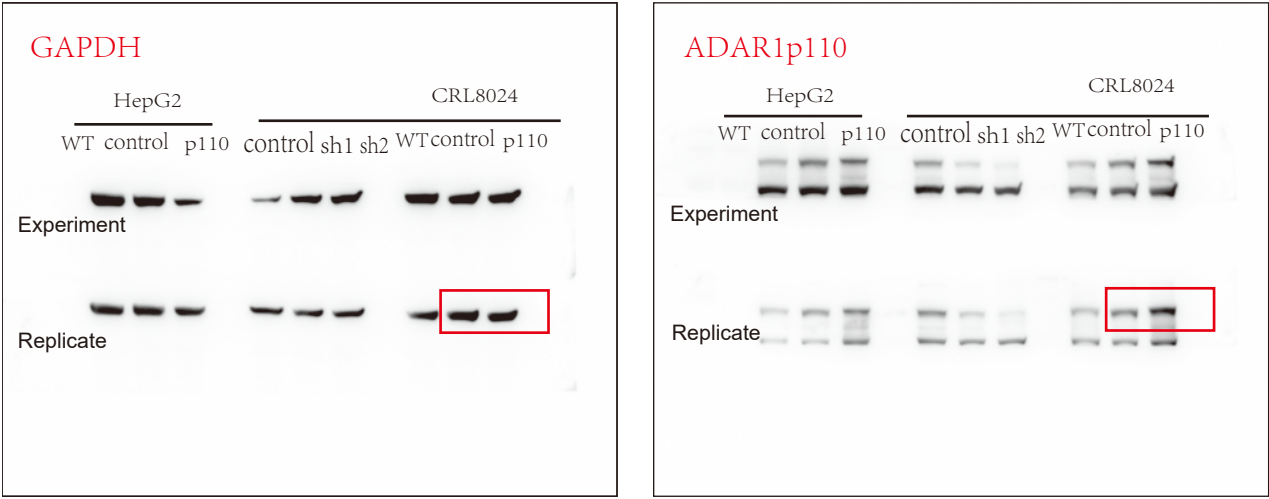

Figure 2B

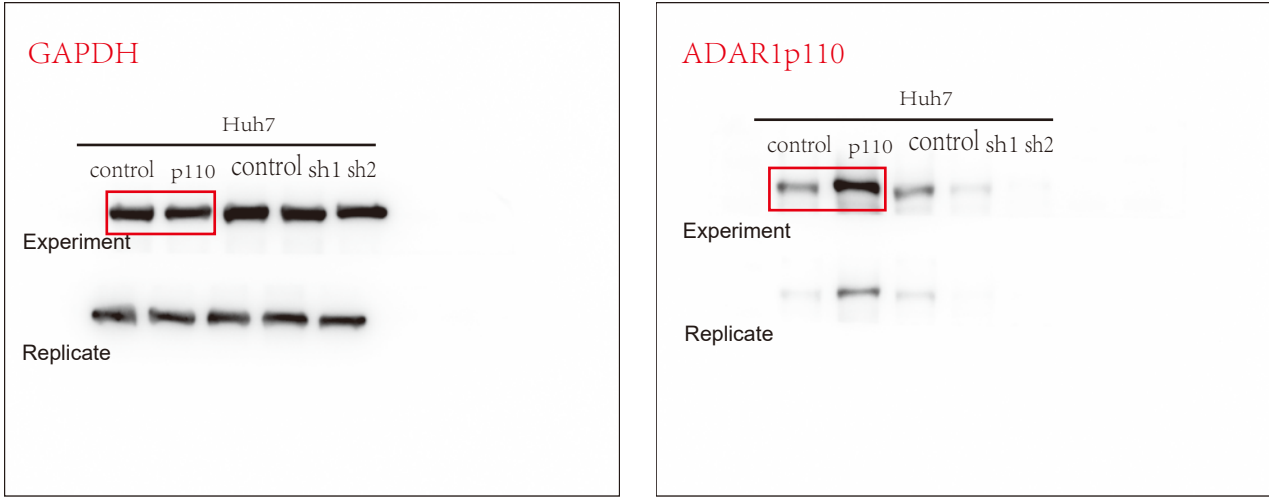

Figure 2G

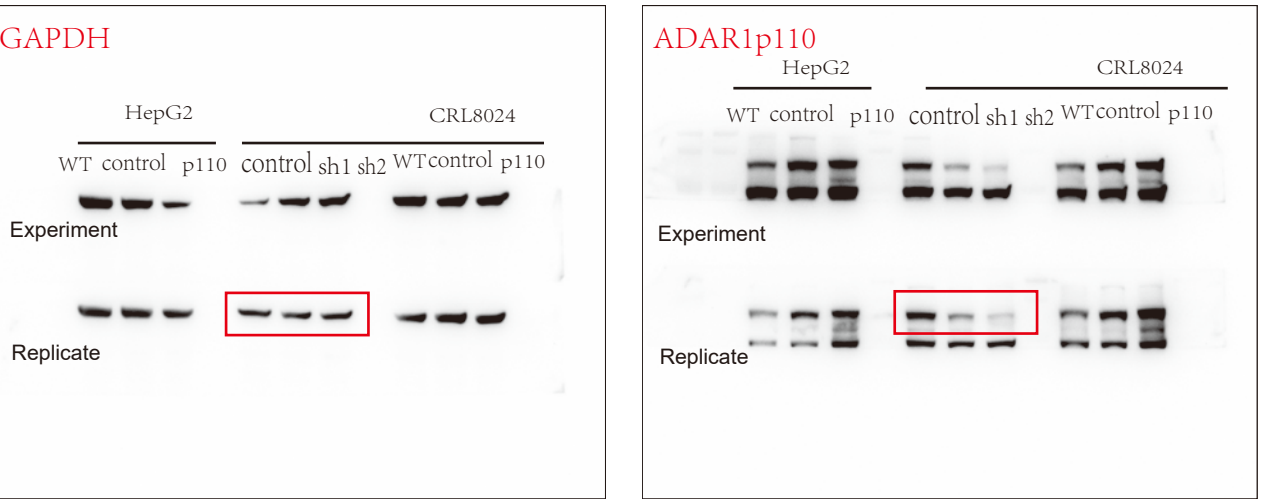

Figure 2I

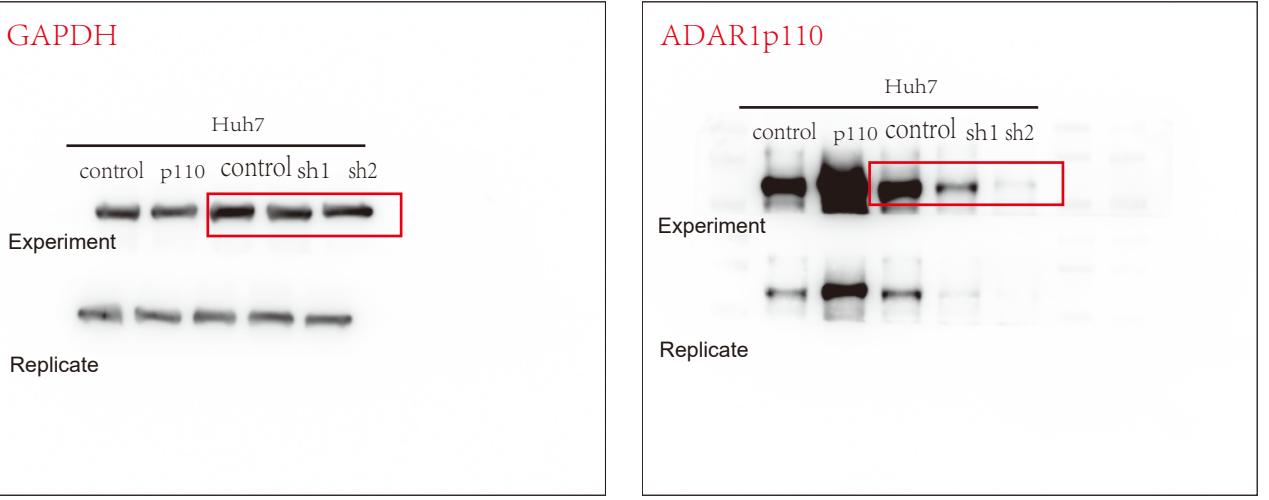

Supplement: Supplementary Figures — Uncropped Western blot images for all panels presented in the main text are provided in Fig. S1 (Fig. 2A, B, G, and H) and S2 (Fig. 6J and K). [file mmc7.pdf]

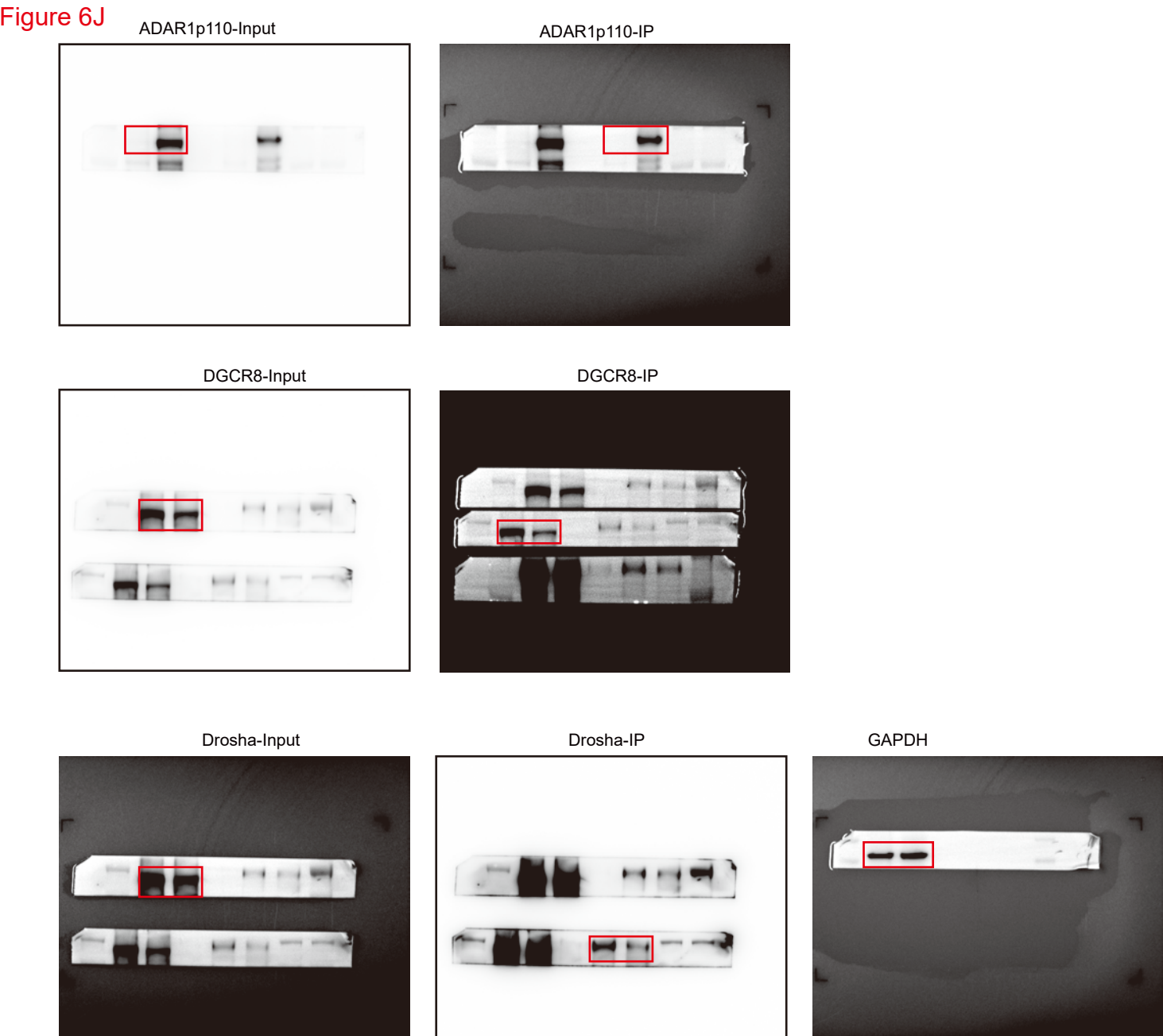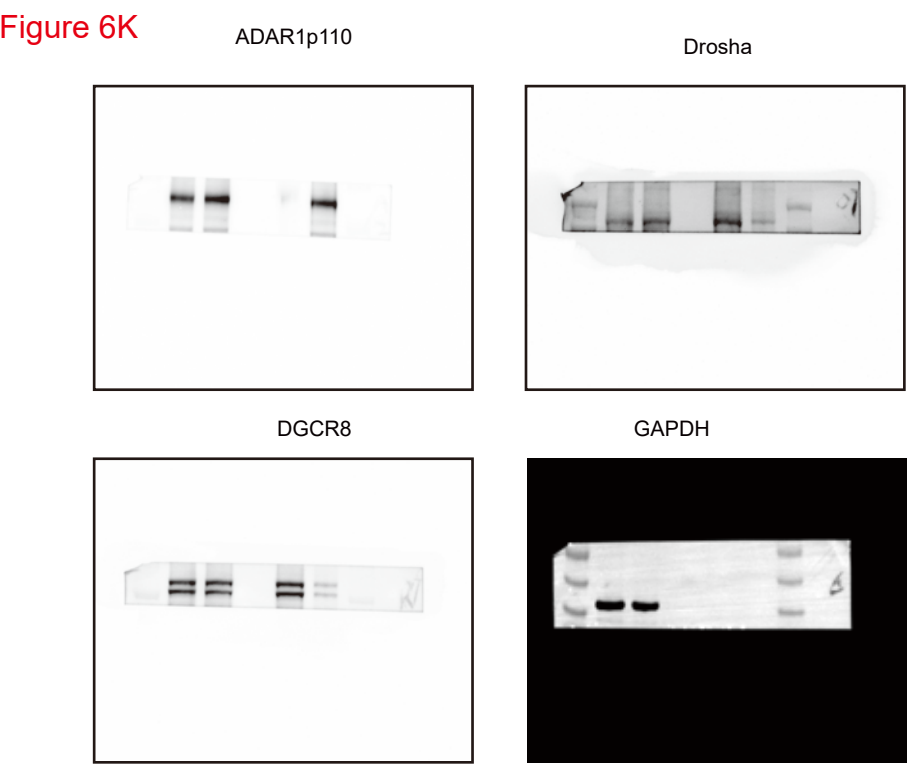

Supplement: Multimedia component 8 [file mmc8.pdf]
